# RAS pathway mutations orchestrate chromatin reconfiguration and transcriptional rewiring in colorectal cancer

**DOI:** 10.1186/s12964-026-03305-0

**Published:** 2026-10-14

**Authors:** Zhangjingyi Gao, Snehangshu Kundu, Pengwei Xing, Eric Cruz Davila, Vamsi Krishna Polavarapu, Ece Akcan, Xingqi Chen, Tobias Sjöblom

**Affiliations:** Science For Life Laboratory, Department of Immunology, Genetics and Pathology, Uppsala University, Uppsala, Sweden

**Keywords:** CRC, RAS pathway mutations, ATAC-seq, WNT signaling, TF, Prognosis

## Abstract

**Background:**

RAS pathway mutations occur in ~ 40% of colorectal cancers (CRCs), with *KRAS-* and *BRAF-* mutant tumors exhibiting distinct mutational, transcriptional, and epigenetic landscapes. Advances in next-generation sequencing enable integrated analyses of multi-omics data, providing insight into their regulatory relationships. To define the molecular programs driven by RAS pathway mutations, we generated isogenic CRC cell models and profiled chromatin accessibility using ATAC-seq.

**Methods:**

We performed ATAC-seq in isogenic HCT116 and RKO models. Mutation-associated changes in genes, regulatory elements, and signaling pathways were identified, together with inferred transcription factor (TF) binding activity. Clinical datasets were analyzed to assess whether features identified in the cell models were recapitulated in patient tumors and whether they were associated with clinical outcomes.

**Results:**

Isogenic CRC models segregated by RAS pathway mutation status at both epigenetic and transcriptomic levels. Mutation alterations modulated WNT signaling, accompanied by changes in RUNX2 and TCF7L2 expression and binding activity. Comparison between the HCT116 and RKO models identified 85 genes, eight of which displayed reproducible expression patterns across three independent clinical cohorts. TCF7L2 were associated with RAS pathway mutations in patient tumors, with elevated expression level predicting worse overall survival independent of cofactors.

**Conclusion:**

We identified RAS pathway mutation–driven regulatory reprogramming with convergent effects on WNT signaling. TCF7L2 emerged as a key TF, with elevated expression in *BRAF*-mutant tumors independently associated with poor clinical outcomes. These findings highlight a clinically relevant epigenetic mechanism linking RAS pathway mutations to CRC cell survival maintenance.

**Graphical abstract:**

**Figure:**
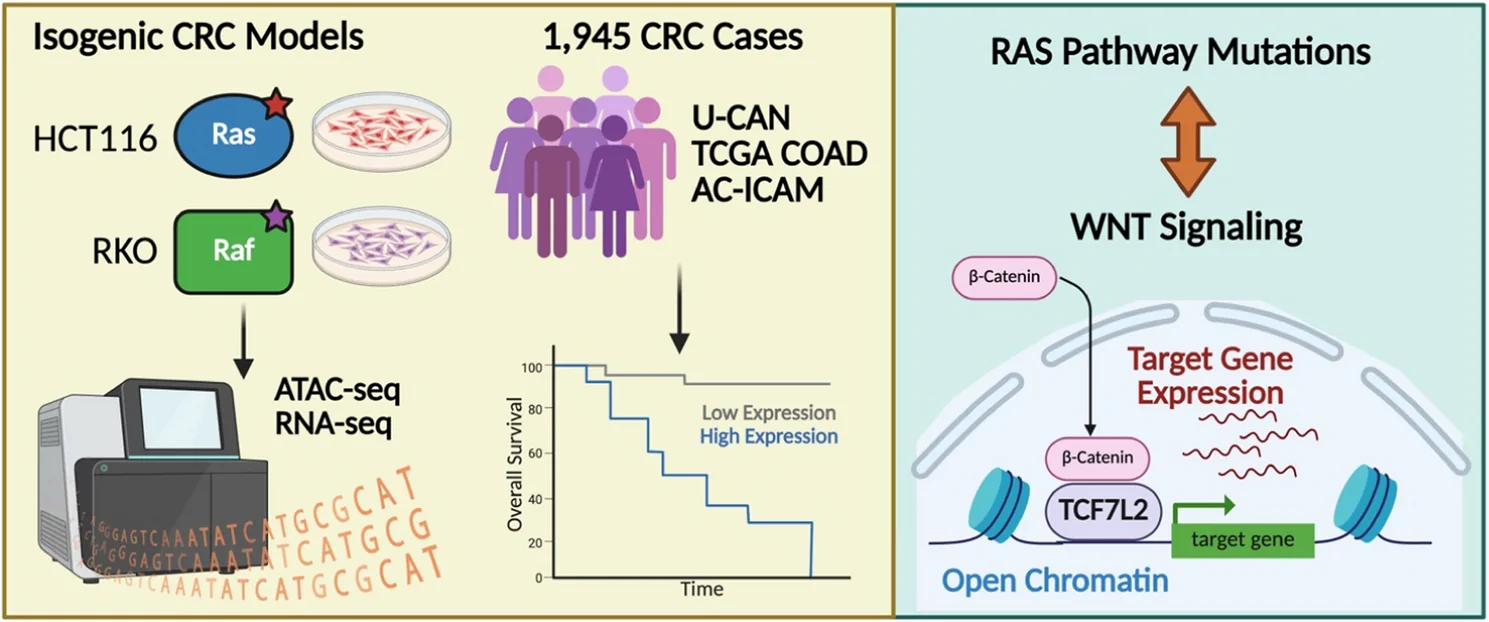

**Supplementary Information:**

The online version contains supplementary material available at https://doi.org/10.1186/s12964-026-03305-0.

## Background

Genome instability and non-mutational epigenetic reprogramming are key features that drive tumor malignancy [1]. Genomic instability promotes tumorigenesis by enabling the accumulation of mutations that activate oncogenic signaling [2], while non-mutational epigenetic reprogramming allows cells to adopt tumor-promoting gene expression programs [3]. Recent study using integrated multi-omics approaches have linked somatic mutations to coordinated changes in gene expression and chromatin accessibility, revealing the reactivation of developmental gene programs driven by TFs [4]. These findings highlight the interplay between genetic alterations, epigenetic landscape, and transcriptional programs, raising the question of how specific oncogenic signals, such as RAS pathway mutations, reshape these molecular profiles.

Somatic RAS pathway mutations are common in CRC, with approximately 40% of cases harboring *KRAS* mutations and 10–20% carrying *BRAF* mutations [4–6]. These tumors exhibit distinct mutational, transcriptional, and epigenetic profiles [6–9]. To explore how RAS mutations contribute to CRC pathogenesis within a controlled framework, a variety of cellular and animal models have been developed. In genome-edited cell models, knockout of the mutant *KRAS* allele in HCT116 cells (retaining the wild-type alleles) reduces anchorage-independent growth, 2D proliferation in vitro and in nude mice, and downregulates *MYC* expression [8]. By contrast, mutant *BRAF* knockout in RKO cells has minimal effects on morphology or proliferation, except for decreased MEK and ERK phosphorylation [9]. A common feature of RAS pathway mutations is enhanced survival under low-glucose conditions, attributed to GLUT1 upregulation—observed in *KRAS*-mutant (HCT116, DLD1) and *BRAF*-mutant (VACO432, RKO) CRC models [10].

To further investigate CRC survival under glucose restriction, we performed a forward genetic screen to identify genes that restore CRC cell growth in low-glucose media following loss of RAS pathway mutations. Stable knockdown of *TCF7L2* and *FOXO3* genes was found to upregulate GLUT1 transcript levels while decreasing anchorage-independent growth in *KRAS*-mutant DLD1 cells [10]. An independent study has found that TCF7L2 regulates GLUT1 and glucose uptake through recruitment of ZIC family TFs [11]. More recently, a CRISPR-based screen in HCT116 cells revealed that glucose restriction protects cancer cells from inhibition of de novo pyrimidine biosynthesis [12]. However, as this screen targeted only ~ 3,000 metabolic genes, the role of additional regulators such as TCF7L2 remains unexplored.

Although both HCT116 and RKO cells are classified as MSI, CIMP+, CMS4 subtype [13], they differ markedly in their dependence on β-catenin-mediated survival. Oncogenic β-catenin activity predominates in HCT116 but not in RKO [14], suggesting distinct interplay between WNT signaling and RAS pathway mutations. Supporting this, small-molecule screening for WNT modulators in CRC showed that MEK1/2 inhibitors enhanced WNT-TOP reporter signal in β-catenin-active cell lines (HCT116, DLD1, SW480) but not in RKO [15]. Furthermore, a genome-scale CRISPR screen uncovered unique epigenetic regulators required for β-catenin-mediated transcription, including KMT2A/MLL1 [16, 17], underscoring the importance of epigenetic context in WNT-driven gene expression.

Building upon these observations, we developed HCT116 and RKO isogenic models to dissect the molecular effects of RAS pathway mutations [18]. Given prior evidence implicating TCF7L2 as a regulator in RAS driven glucose metabolism [10, 11] and its reliance on chromatin accessibility [17], we hypothesized that epigenetic mechanisms underlie the transcriptional reprograms of RAS pathway mutation alterations.

## Methods

### Isogenic model construction

All isogenic clones were generated by recombinant AAV-mediated homologous recombination (knock in/out at the endogenous locus). The detailed protocol has been described in our previous work [18]. To ensure the targeting constructs matched the cell line’s own genomic background and avoid introducing unintended SNPs, homology arm 1 (HA1) for each construct was PCR-amplified directly from genomic DNA of the parental cell line rather than from a standard reference template. The desired *KRAS* or *BRAF* point mutation was then incorporated into HA1 by stitching PCR using mutagenic primers. Gateway cloning was used to assemble the mutant HA1 together with the remaining homology arm and the selection cassette into the final destination-vector targeting construct. The clones were derived as follows: Parental HCT116 carries one mutant and one wildtype *KRAS* allele. DK clones were derived from HCT116 by knocking out the mutant *KRAS* allele, leaving a single wildtype *KRAS* allele. MHB clones were derived from DK by knocking in one mutant *BRAF* allele, resulting in one wildtype and one mutant *BRAF* allele. Parental RKO carries two mutant and one wildtype *BRAF* allele. RB clones were derived from RKO by knocking out both mutant *BRAF* alleles, leaving a single wildtype *BRAF* allele. MH (*KRAS* G13D), MHC (*KRAS* G12C), MHV (*KRAS* G12V) and MHD (*KRAS* G12D) were derived from RB by knocking in one hotspot mutant *KRAS* allele, leaving one mutant and one wildtype *KRAS* allele.

### Cell lines and cell culture

HCT116 and RKO cells were cultured in McCoy’s 5 A medium in T75 flasks until approximately 80% confluence. Cells were then trypsinized and dissociated into a single-cell suspension. Following a single wash with PBS/HBSS, at least 100,000 cells per sample were counted and collected as fresh cell pellets. Three to four biological replicates were prepared per clone.

### Library preparation with Omni ATAC protocol and sequencing

Omni-ATAC was performed as previously described [19, 20]. Briefly, 50,000 cells per sample were permeabilized using NP-40, Tween-20, and digitonin to reduce mitochondrial DNA contamination. Cells were first centrifuged at 500×*g* for 5 min at 4 °C in a fixed-angle microcentrifuge, then resuspended in 50 µL buffer (10 mM Tris-HCl, pH 7.4; 10 mM NaCl; 3 mM MgCl₂; 0.1% NP-40; 0.1% Tween-20; and 0.01% digitonin) and incubated on ice for 30 min. Following incubation, 950 µL of the same buffer was added, and cells were centrifuged again at 500×g for 10 min at 4 °C. Cell pellets were gently resuspended in the transposase reaction mix, which included 12.5 µL 2× transposase buffer, 16.5 µL 1× PBS, 0.5 µL 1% digitonin, 0.5 µL 10% Tween-20, 2.5 µL Tn5 transposase, and 5 µL nuclease-free water, totaling 37.5 µL. The reaction was incubated at 37 °C for 30 min. Transposed DNA was purified using MinElute PCR Purification Kit (Qiagen, Cat# 28004) and eluted in 10 µL of Qiagen EB elution buffer. Sequencing libraries were then prepared according to the original ATAC-seq protocol [21] and submitted to Novogene.

### ATAC-seq data processing and quality control analyses

Adapter-trimmed reads were aligned to GRCh38 by Bowtie2 [22]. Reads mapping to mitochondrial, with MAPQ < 10 were removed by SamTools. PCR duplicates were eliminated by MarkDupliates.jar (http://broadinstitute.github.io/picard/). Tn5 transposase offsets were corrected (+ 4/-5 bp). Peaks were called using MACS2 [23] (q < 0.01). Peaks overlapping ENCODE blacklisted regions [24] were excluded. Peaks were merged across samples using BedTools [25], followed by read counts recalculated and low-abundance peaks (bottom 5%) removed. Data were normalized to counts per million (CPM) using EdgeR. Replicate consistency was assessed by PCA and Pearson correlation.

### Differential analysis by Deseq2 with ATAC-seq and RNA-seq data

Differential analysis were performed using DESeq2 [26], with comparisons conducted between RAS pathway mutations and their relative wild-type. Significance thresholds were adjusted *p* < 0.05 and |log2 Foldchange| > 0.58.

### Genomic annotation

ATAC-seq peaks were annotated using ChIPseeker [27]. Promoters were defined as -1 kb to + 100 bp relative to the TSS. Distal regulatory elements were defined as regions > 3 kb from the TSS and within 500 kb. Peaks were assigned to promoter, UTR, exon, intron, or intergenic categories.

### Functional enrichment analysis

GO enrichment analysis was performed using clusterProfiler [28] with MSigDB C5 Biological Process gene sets. Significance terms (BH adjusted p-value < 0.05) were clustered by sematic similarity by simplifyEnrichment. Genes were ranked by RNA-seq log2Foldchange to conduct GSEA analysis using Hallmark gene sets.

#### TF analysis

TF binding to regulatory regions was evaluated using chromVAR [29] with JASPAR motifs and GRCh38 genome annotations. Peaks with variability scores > 1 were retained, and differential motif accessibility between RAS mutation group was assessed using Student’s *t*-tests with BH correction (adjusted *p* < 0.05). TF footprinting was performed using TOBIAS [30]. BAM files were merged per clone, Tn5 insertion bias corrected, and footprinting scores computed. Differential TF binding was identified using BINDetect, and peaks were annotated with UROPA based on GENCODE v45 gene annotations. TFs were retained on concordant chromVAR deviations and extreme (top/bottom 5%) footprinting scores.

### Clinical cohort analysis

For the U-CAN cohort [6], bulk RNA-seq data and clinical metadata were obtained from the CNGB Sequence Archive (CNSA; accession E-MTAB-12862), and somatic coding-region variant calls were downloaded from the European Variation Archive (accession RPJEB61514). Patients harboring *BRAF* V600E or *KRAS* hotspot mutations (G13D, G12C, G12V, G12D) were identified using ClinVar annotations. For the AC-ICAM cohort [31], bulk RNA-seq data, clinical annotations, and mutation status were obtained from cBioPortal. For the TCGA COAD cohort, bulk RNA-seq, bulk ATAC-seq, clinical data, and mutation information were downloaded from GDC Data Portal. Gene expression differences between mutation groups were evaluated using Wilcoxon rank-sum tests (*p* < 0.05). The individual effects of *BRAF* and *KRAS* mutations were further assessed using one-way ANOVA followed by Tukey’s HSD post hoc tests. TF analyses in clinical cohorts were performed using the same approach descried above.

### Survival analysis

Overall survival was defined as the time from primary tumor diagnosis to death or last follow-up. Patients with available RAS pathway mutation status were included in the survival analyses [6]. Cox proportional hazards models were employed to evaluate associations between expression and survival using the survival and survminer R packages. Univariate Cox regression was performed for all candidate genes, and genes with *p* < 0.05 were included in multivariate models adjusting for co-factors. Genes significant in univariate analyses were used to conduct log-rank tests and generate Kaplan-Meier survival curves, with patients stratified expression levels using the 75th percentile cutoff.

### Western blot

Cells were harvested and lysed in RIPA buffer (B9901, Thermo Fisher Scientific) containing protease and phosphatase inhibitors (PPC1010, Sigma-Aldrich) for 30 min on ice. Total protein concentrations were quantified using the BCA assay (23225, Thermo Fisher Scientific). A total of 20 µg of protein was denatured with NuPAGE LDS buffer and reducing agent at 95 °C for 5 min. Protein samples were resolved on a NuPAGE 4–12% Bis-Tris gel. Samples were then transferred onto a 0.2 μm pore-size nitrocellulose membrane. Membranes were blocked in Tris-buffered saline with 0.1% Tween-20 (TBS-T) and 5% BSA for 1 h at room temperature. Membranes were sequentially probed with phosphorylated-ERK1/2 and ERK1/2 primary antibodies (4370/4695, Cell Signaling Technology) at 4 °C overnight. Western blot stripping buffer (46430, Thermo Fisher Scientific) was used according to the manufacturer’s instructions before reprobing membranes with β-actin primary antibody (sc-47778, Santa Cruz Biotechnology) for 1 h at room temperature. Following primary antibody incubation, membranes were washed three times with TBS-T for 5 min each before incubating with the appropriate secondary antibody for 1 h at room temperature. Membranes were then washed as previously mentioned before chemiluminescent detection was performed using SuperSignal West Pico PLUS (34577, Thermo Fisher Scientific).

## Results

**Fig. 1:**
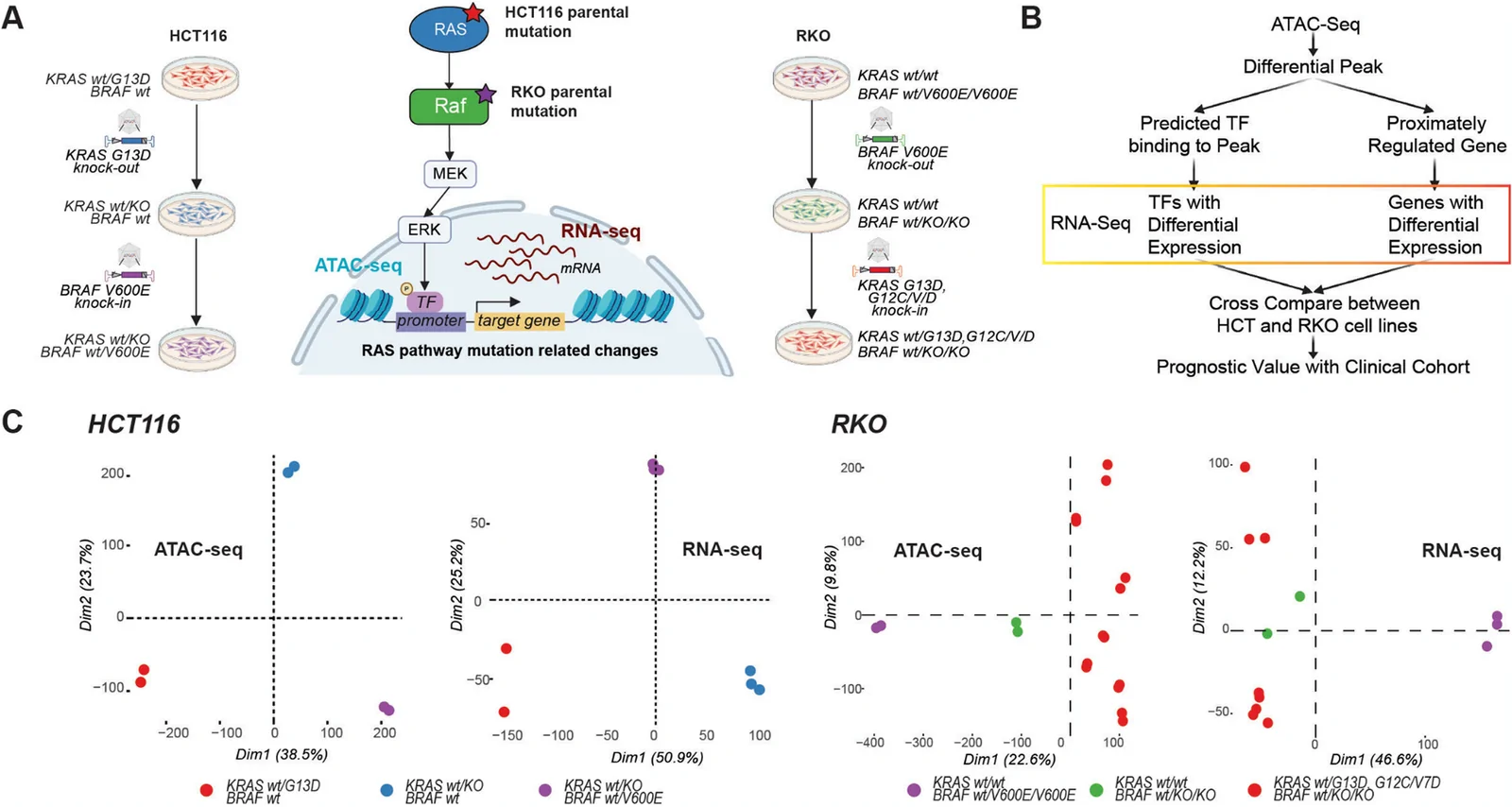
RAS pathway mutation alteration leads to distinct transcriptomic and epigenetic profiles. **A** Isogenic models of RAS pathway mutations. The *KRAS* G13D mutation (red) was knocked out in HCT116 parental clones to generate RAS pathway wild-type clones (blue), followed by introduction of the *BRAF* V600E mutation (purple). The *BRAF* V600E mutation (purple) was knocked out in RKO parental clones to generate RAS pathway wild-type clones (green), followed by introduction of *KRAS* hotspot mutations (red). The cell models were generated in previous work [18]. **B** Overview of the multi-omics analysis workflow. **C** PCA of ATAC-seq and RNA-seq data shows stratification by RAS pathway mutation in HCT116 (left) and RKO (right) cell clones

To understand how mutations in the RAS pathway alter the transcriptional and epigenetic profiles, we analyzed isogenic HCT116 and RKO models [18] (Fig. 1A).

### RAS pathway mutation alteration leads to distinct transcriptomic and epigenetic profiles

To assess epigenetic profiles under different RAS mutation status, ATAC-sequencing was performed and integrated with previously generated RNA-sequencing data [18] (Fig. 1B). Data quality was evaluated based on total raw read counts, fraction of reads in peaks (FRiP scores), and transcription start site (TSS) enrichment scores. All HCT116 and RKO clones were confirmed to have high-quality data (Supplementary Fig. 1B-D). Principal component analysis (PCA) and pairwise Pearson correlation was applied to global chromatin accessibility and gene expression profiles to examine reproducibility among RAS pathway mutation replicates. Distinct landscapes were revealed to be shaped by RAS pathway mutations (Fig. 1C), with markable alterations observed following the removal of parental mutations. The RKO clones harboring *KRAS* hotspot mutations (G13D, G12D, G12C, and G12V) clustered closely along the first principal component (Fig. 1C) and were therefore grouped into a single *KRAS* mutant category for downstream analyses. Pairwise Pearson correlation matrix further confirmed the most divergency is between parental RAS-mutant clones and their derivative clones, whereas minimal changes were detected after introducing *KRAS* mutations into RAS wild-type RKO clones (Supplementary Fig. 1E, Supplementary Fig. 1F). No differences were observed in cis-regulatory element (CRE) features assigned to globally regulatory regions across RAS pathway mutations (Supplementary Fig. 1G).

Overall, quality control supported the validity of our isogenic cell line models for studying mutations-driven epigenetic and transcriptional landscapes.

### RAS pathway mutations modulate the WNT signaling pathway at both transcriptomic and epigenetic levels

We next aimed to identify genes and regulatory regions that are associated with RAS pathway mutations. To this end, we performed pairwise comparisons between RAS-mutant and their corresponding wild-type clones.

**Fig. 2:**
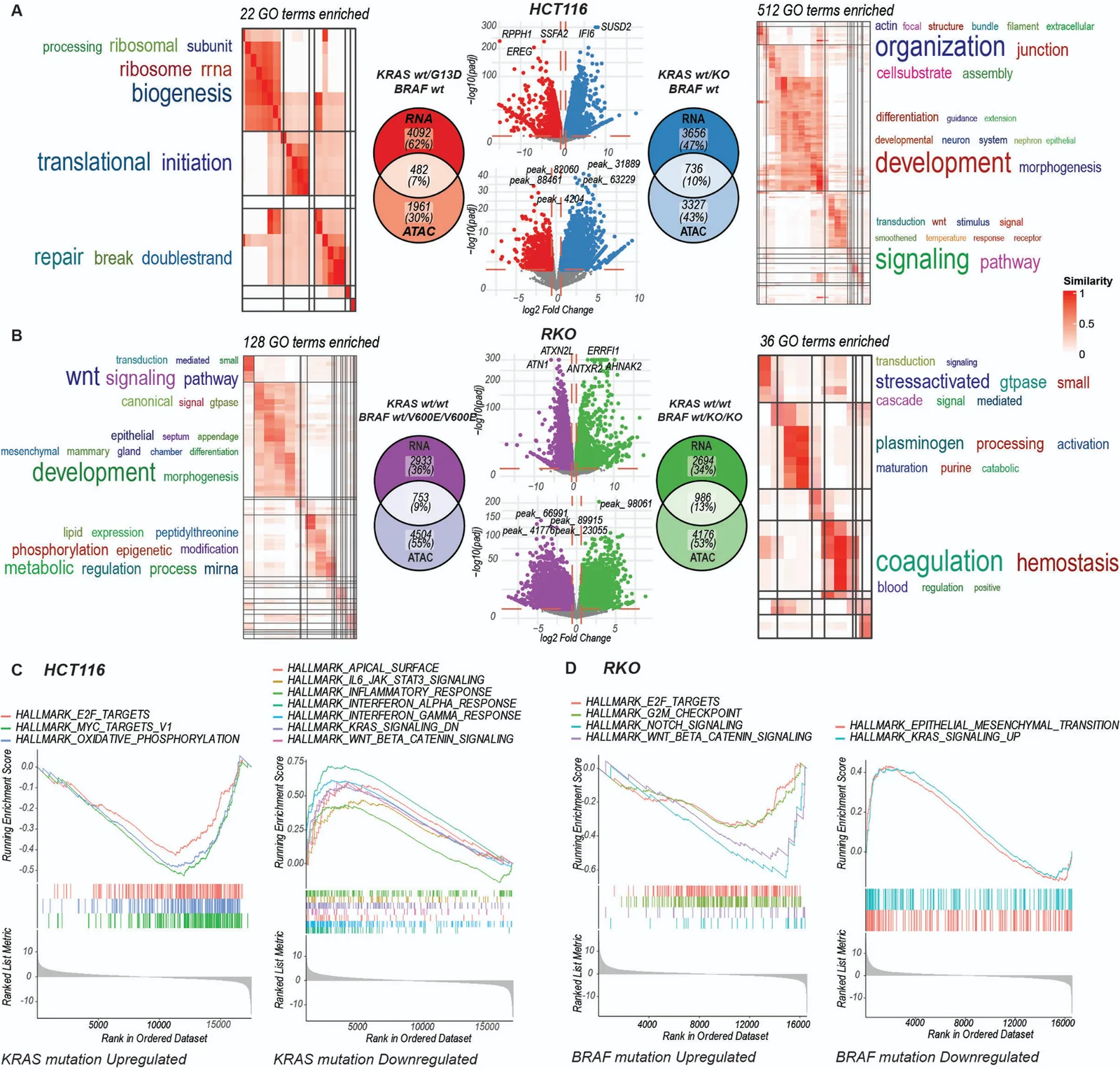
RAS pathway mutations modulate the WNT signaling pathway at both transcriptomic and epigenetic levels. **A** Volcano plot displays *KRAS* mutation-associated genes and regulatory regions in HCT116 clones (adjusted *p* < 0.05; |log2FC| > 0.58). Venn diagrams show intersecting genes between RNA-seq (top) and ATAC-seq (bottom). Heatmaps depict GO term clusters of intersecting genes (adjusted *p* < 0.05), with word clouds summarizing keywords for each cluster. **B ***BRAF* mutation-associated genes and regulatory regions in RKO clones (adjusted *p* < 0.05; |log2FC| > 0.58), with enriched biological functions (adjusted *p* < 0.05). **C** Transcriptional hallmark pathways regulated by *KRAS* mutation in HCT116 clones. Gene set enrichment analysis (GSEA) was performed using all expressed genes ranked by log2FC (adjusted *p* < 0.05). Pathways are shown as *KRAS* mutation upregulated (left) and downregulated (right). **D** Transcriptional hallmark pathways regulated by *BRAF* mutation in RKO clones (adjusted *p* < 0.05), with upregulated (left) and downregulated (right) pathways

Knockout of the *KRAS* mutant allele in parental HCT116 clones resulted in 3,656 upregulated and 4,092 downregulated genes (*adjusted p < 0.05; |log2FC| > 0.58*), along with 3,327 upregulated and 1,961 downregulated regions (*adjusted p < 0.05; |log2FC| > 0.58*). Among these, 736 genes were consistently upregulated and 482 were downregulated at both the transcriptomic and epigenetic levels (Fig. 2A). To explore the biological relevance of these intersecting genes, gene ontology (GO) enrichment analysis was conducted. Five hundred and ten GO terms were enriched from the upregulated set with clusters primarily associated with cellular organization, development, and signaling pathways. In contrast, 22 GO terms were enriched from the downregulated set, mainly related to ribosome biogenesis, translational initiation, and double strand break repair (Fig. 2A).

Similarly, knockout of the *BRAF* mutant alleles in RKO parental clones resulted in 2,694 upregulated and 2,933 downregulated genes (*adjusted p < 0.05; |log2FC| > 0.58*), alongside 4,176 upregulated and 4,504 downregulated regions (*adjusted p < 0.05; |log2FC| > 0.58*). Of these, 986 genes were consistently upregulated and 753 were downregulated at both transcriptomic and epigenetic levels (Fig. 2B). GO analysis revealed 36 enriched terms from the upregulated intersecting genes, focusing on stress-activated and small GTPase signal transduction, plasminogen activation, and coagulation/hemostasis, while 128 terms were enriched from the downregulated intersecting genes, predominantly associated with signaling pathways-particularly WNT signaling, as well as development and epigenetic and metabolic regulation processes (Fig. 2B). The GO term “epigenetic regulation of gene expression” was specifically enriched within *BRAF*-mutant RKO clones, providing direct evidence that *BRAF* mutations modulate global epigenetic profiles. There are 19 genes enriched in the term, but *KMT2A* and *ARID1A* caught our interest, as *KMT2A* has been previously reported to regulate endogenous transcription beyond the canonical TCF4/TCF7L2 in β-catenin dependent cells [16]. Meanwhile, *ARID1A*, which shows a relatively high mutation frequency of 16%^6^, whose loss has been reported to impair *KRAS*-mutant CRC growth, with a notable strong colocalization between ARID1A and TCF7L2 [17].

Since signaling pathways emerged as a prominent cluster, with WNT signaling standing out, we performed GSEA on ranked RNA-seq data to further identify the enriched pathways. Consistent with GO results, WNT signaling was enriched following *KRAS* mutation knockout in HCT116 clones (Fig. 2C), while enriched prior to *BRAF* mutation knockout in RKO clones (Fig. 2D). KRAS signaling was downregulated after *KRAS* mutation knockout in HCT116 clones (Fig. 2C), reflecting *KRAS* mutation’s role as a direct oncogenic driver [6]. In contrast, KRAS signaling was upregulated after *BRAF* mutation knockout in RKO clones (Fig. 2D), likely due to the release of ERK-mediated feedback inhibition imposed by the *BRAF* V600E mutation, which functions as a RAS-independent monomer that suppresses RAS via hyperactivated ERK [32]. Of note, MYC signaling was downregulated, consistent with decreased *MYC* gene expression and reduced chromatin accessibility in promoter regions. Meanwhile, immune response pathways, including inflammatory and interferon responses, were upregulated in HCT116 clones after *KRAS* mutation knockout aligning with reports that *KRAS* mutation impairs the IFN response in CRC by suppression of STAT3-driven DDX60 transcription [33].

Introduction of novel RAS pathway mutations into HCT116 and RKO wild-type clones revealed fewer gene and chromatin accessibility changes compared to the removal of parental mutations. Specifically, introduction of *BRAF* V600E in HCT116 clones led to 557 downregulated and 247 upregulated genes (Supplementary Fig. 2A), with WNT signaling downregulated (Supplementary Fig. 2B). Conversely, introduction of the *KRAS* hotspot mutations (*G13D*,* G12 C/V/D*, analyzed collectively as described above) in RKO clones yielded 76 downregulated and 199 upregulated genes (Supplementary Fig. 2C), with no WNT associated changes detected. Instead, TGF-β signaling emerged before *KRAS* hotspot mutation introduction. Gene set enrichment analysis revealed KRAS signaling was downregulated before novel mutation introduction in HCT116 clones (Supplementary Fig. 2D), whereas both upregulation and downregulation of KRAS signaling was observed following novel mutation introduction in RKO clones (Supplementary Fig. 2E), suggesting that it is dynamically modulated by the acquisition of RAS pathway mutations. Moreover, MYC target pathway was restored following *BRAF* mutation introduction into HCT116 clones in parallel with increased *MYC* gene expression.

To validate these transcriptomic changes at the protein level, we performed immunoblotting for phosphorylated and total ERK1/2 in HCT116 and RKO clones (Supplementary Fig. 9A, B). Consistent with the release of ERK-mediated feedback and reduced MAPK pathway output following *BRAF* mutation knockout, relative p-ERK level decreased in RAS wildtype RKO clones, while introduction of BRAF mutation into RAS wildtype HCT116 clones significantly increased p-ERK level (*p* = 0.0051 vs. wildtype; *p* = 0.0331 vs. *KRAS* G13D). Expression of MAPK pathway-related genes (*DUSP*, *MEK* and *ERK*) was correspondingly altered across HCT116 and RKO clones (Supplementary Fig. 9C). This indicates *BRAF* V600E mutation may act as an independent driver of MAPK pathway activation.

Taken together, our findings demonstrate that RAS pathway mutations modulate WNT signaling at both transcriptomic and epigenetic levels.

### Transcription factors RUNX2 and TCF7L2 are key TFs under RAS pathway mutation alterations

To further investigate the feature of regulatory regions, we first mapped them back to the reference genome and classified based on proximity to TSS. TF binding activity was then inferred from regulatory region changes.

**Fig. 3:**
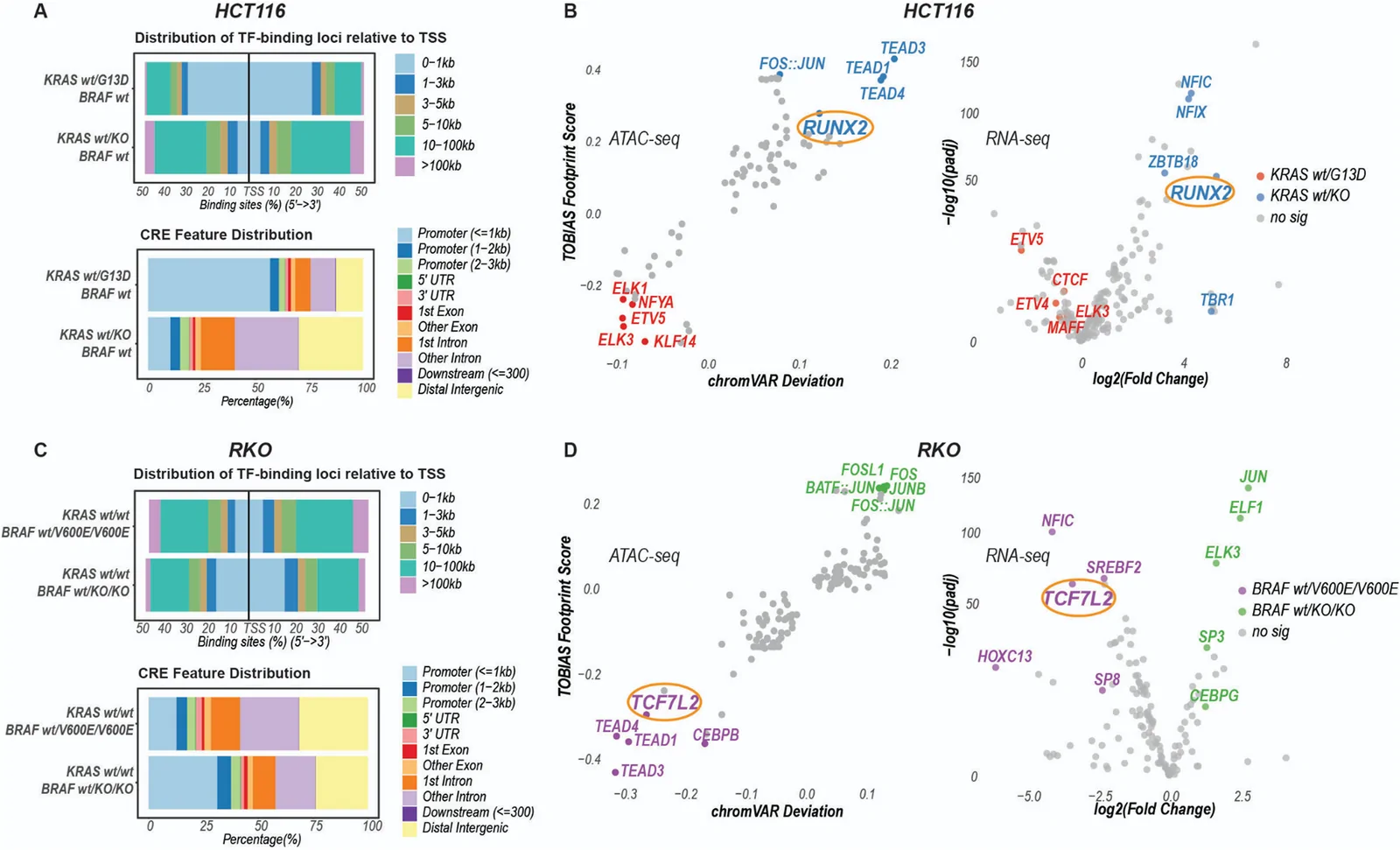
RUNX2 and TCF7L2 emerge as top TFs regulated by RAS pathway mutations. **A ***KRAS* mutation-associated CREs identified in HCT116 clones. Top bar plot, genomic distribution of TF-binding loci relative to TSS (-50 kb to + 50 kb). Bottom bar plot, CRE feature assignments of TF-binding loci, with promoters defined as 0–3 kb proximately to TSS. **B ***KRAS* mutation-associated TFs predicted by integrating binding loci accessibility and expression levels in HCT116 clones. Scatter plot (left) shows TF binding activity predicted using transcription factor occupancy prediction by ATAC-seq (TOBIAS; *adjusted p < 0.05*) and chromatin variation across regions (chromVAR; *variability > 1*,* t-test adjusted p < 0.05*). Top 5 TFs for each RAS pathway mutation type are highlighted. Volcano plot (right) shows TF expression levels upon RAS pathway mutation (*adjusted p < 0.05*), with top 5 TFs highlighted. **C ***BRAF* mutation-associated CREs annotated. **D ***BRAF* mutation-associated TFs predicted by binding site accessibility (TOBIAS, *adjusted p < 0.05;* chromVAR, *variability > 1*,* t-test p < 0.05*) and expression integration (*adjusted p < 0.05*)

Following *KRAS* mutation knockout in HCT116 clones, 60% of downregulated regions were promoters, whereas only < 20% in upregulated set (Fig. 3A). To identify which TF bind to these cis regulatory elements (CREs), we applied TF enrichment approach chromVAR and TF footprint approach TOBIAS. Significant TFs were identified in HCT116 clones followed *KRAS* mutation alterations (Supplementary Fig. 3A, C). Cross-referencing with RNA-seq data highlighted TFs whose expression changes were concordant with binding activity. Notably, RUNX2 and TEAD family members demonstrated strongest enrichment in binding activity following *KRAS* mutation knockout, with RUNX2 additionally upregulated in expression (Fig. 3B). In RKO clones, 20% of downregulated regions following *BRAF* mutation knockout were in promoters, compared with 40% in upregulated set (Fig. 3C). Significant TFs were identified in RKO clones followed *BRAF* mutation alterations (Supplementary Fig. 3B, D). TCF7L2 and TEAD family members were enriched in parental RKO clones, with TCF7L2 showing reduced expression after *BRAF* mutation knockout (Fig. 3D). Introduction of novel RAS pathway mutations into wild-type clones recapitulated similar chromatin remodeling patterns. Both HCT116 (Supplementary Fig. 4A) and RKO (Supplementary Fig. 4C) clones showed decreased promoter proportions. Significant TFs were identified in both models (Supplementary Fig. 5). RUNX2 and TCF7L2 were enriched after introduction of *BRAF* mutations into wild-type HCT116 clones (Supplementary Fig. 4B).

Taken together, these findings indicate that RAS pathway mutations reshape chromatin accessibility at promoter regions, leading to coordinated changes in TF binding and expression, including RUNX2 and TCF7L2.

### RUNX2 and TCF7L2 regulate WNT signaling genes upon RAS pathway mutation alteration

To evaluate whether the identified TFs regulate genes associated with RAS pathway mutations, we queried the DoRothEA TF regulon database using mutation-associated TFs as sources and genes identified by both ATAC-seq and RNA-seq as targets (Supplementary Fig. 6). Notably, changes of both RUNX2 and TCF7L2 align with the regulatory direction of WNT signaling, which suggests their potential role as regulator behind.

**Fig. 4:**
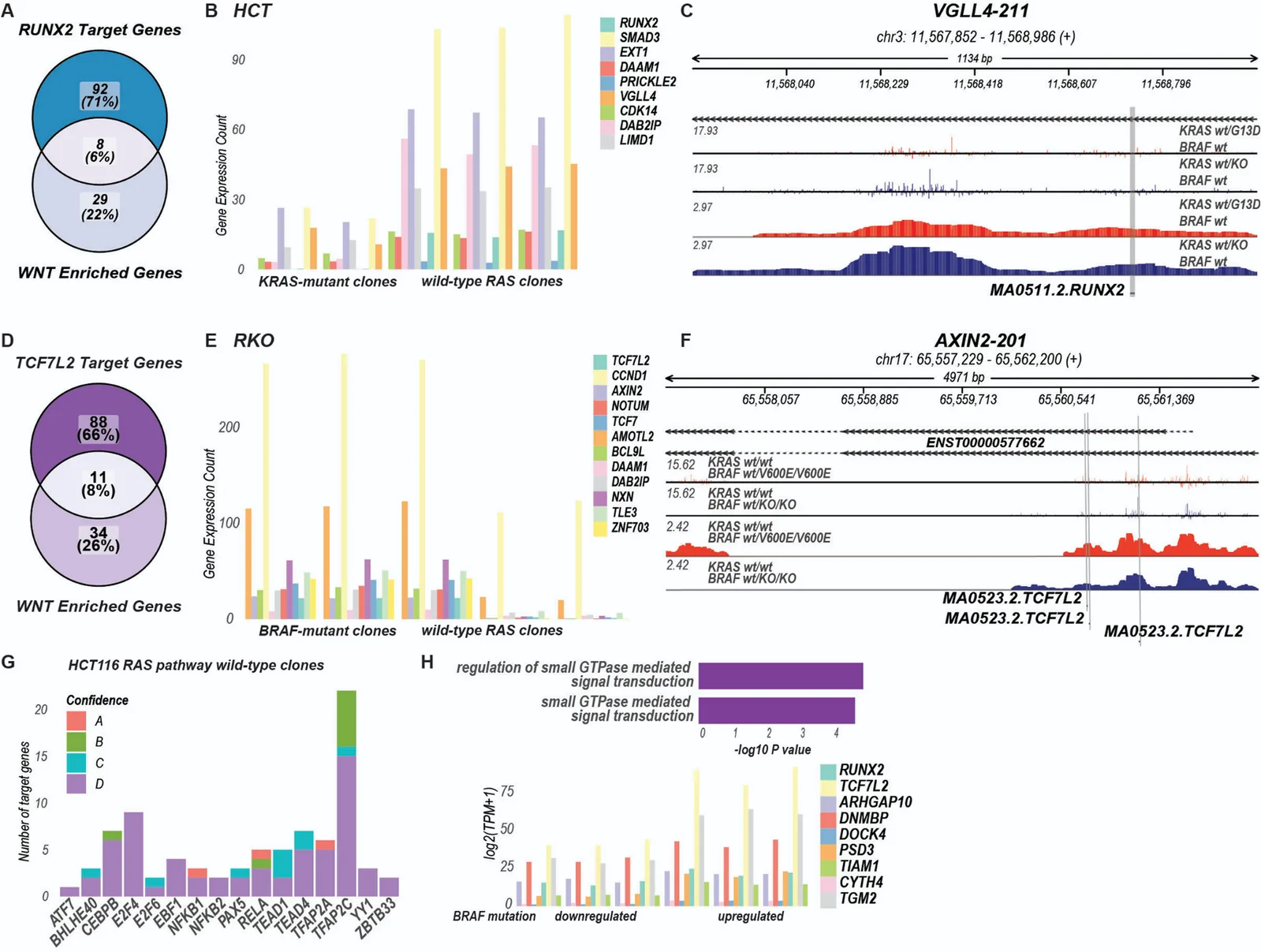
RUNX2 in HCT116 and TCF7L2 in RKO regulate WNT signaling genes upon RAS pathway mutation. **A** Venn diagram of RUNX2-regulated WNT pathway genes in wild-type RAS HCT116 clones. RUNX2 target genes (top) were derived from the DoRothEA TF regulon collection and overlapped with intersecting gene set. WNT-enriched genes (bottom) were identified from the GO term *WNT signaling pathway*. **B** Expression of RUNX2 and its WNT target genes in HCT116 clones. **C** Genomic track plot showing RUNX2 footprints predicted in the *VGLL4* promoter region by TOBIAS in HCT116 clones. **D** Venn diagram of TCF7L2-target WNT pathway genes in *BRAF*-mutant RKO clones. **E** Expression of TCF7L2 and WNT target genes in RKO clones. **F** Genomic track plot showing TCF7L2 footprints predicted in the *AXIN2* promoter by TOBIAS in RKO clones. **G** TFs associated with WNT signaling alteration following *BRAF* mutation reintroduction in HCT116 clones. RAS pathway mutation target genes regulated by TFs were intersected with WNT signaling genes (adjusted *p* < 0.05) to identify TFs that involves in WNT pathway regulation. **H** Following *BRAF* mutation reintroduction, RUNX2 and TCF7L2 were found to regulate genes involved in small GTPase-mediated signal transduction in HCT116 clones. The bar plot (above) shows GO terms enriched among genes coregulated by RUNX2 and TCF7L2 (adjusted *p* < 0.05). The bar plot (below) shows expression changes in these genes

From 37 genes annotated to the WNT signaling GO term, we intersected with 100 RUNX2 target genes and identified 8 overlapping genes: *SMAD3*,* EXT1*,* DAAM1*,* PRICKLE2*,* VGLL4*,* CDK14*,* DAB2IP*, and *LIMD1* (Fig. 4A-B). Since these target genes have at least ChIP-seq level evidence, we applied TOBIAS to predict RUNX2 binding at their regulatory regions. A clear RUNX2 binding site was identified in the *VGLL4* promoter (Fig. 4C). Similarly, intersecting 45 WNT signaling genes with 99 TCF7L2 target genes revealed 11 overlapping genes: *CCND1*,* AXIN2*,* NOTUM*,* TCF7*,* AMOTL2*,* BCL9L*,* DAAM1*,* DAB2IP*,* NXN*,* TLE3*, and *ZNF703* (Fig. 4D-E). TCF7L2 binding sites were predicted within the *AXIN2* promoter by TOBIAS (Fig. 4F). Since WNT signaling was also upregulated in HCT116 RAS wild-type clones prior to introducing *BRAF* mutations, while TCF7L2 and RUNX2 emerged in the opposite direction, we next investigated the TFs potentially regulating WNT signaling (Fig. 4G), as well as the pathway governed by TCF7L2 and RUNX2. Two enriched terms were identified: regulation of small GTPase mediated signal transduction and small GTPase mediated signal transduction (Fig. 4H).

### Genes consistently regulated by RAS pathway mutation in clinical cohorts

**Fig. 5:**
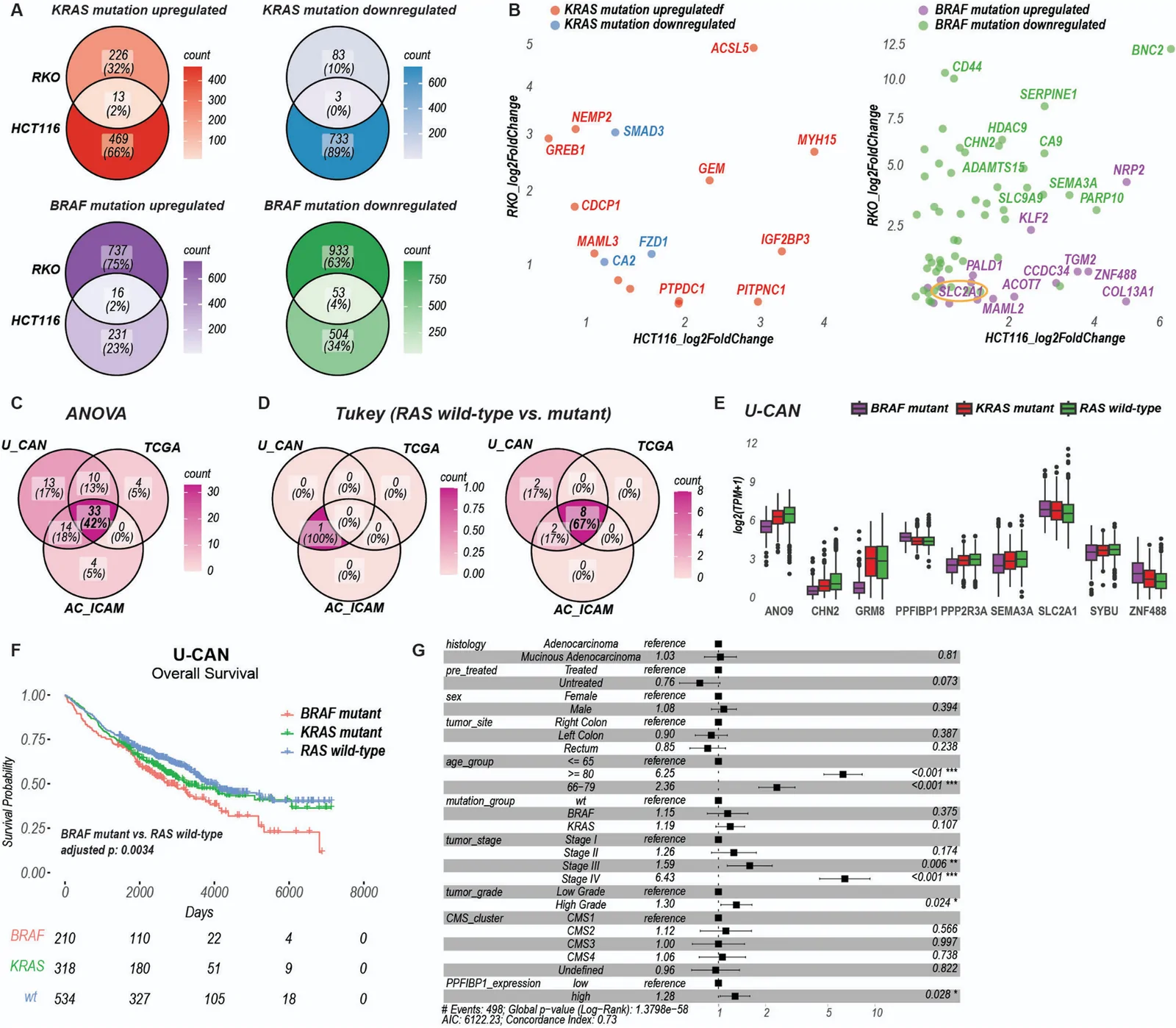
One-third of genes altered upon RAS pathway mutation from cell models also show mutation-dependent expression pattern in human CRCs. **A** Venn diagram of RAS pathway mutation-altered genes captured in both HCT116 and RKO cell models. **B** Scatter plot of expression levels for mutation-altered genes, with the top 10 most altered genes labeled. **C** Venn diagram of cell model reported genes that show mutation-dependent expression pattern in the U-CAN, TCGA, and AC-ICAM cohorts (*ANOVA test*,* p* < 0.05). **D** Venn diagram of genes showing consistent regulation direction in clinical cohorts compared to cell models (*Tukey test*, *RAS pathway wildtype vs. mutant*,* p* < 0.05). **E** Expression of genes reported consistent regulation in the U-CAN cohort, with *SLC2A1* shown as a positive control. **F** KM overall survival plot stratified by RAS pathway mutation in U-CAN cohort. **G** Forest plot of multivariate analysis for *PPFIBP1* expression group in U-CAN cohort

The use of two isogenic cell lines was intended to capture RAS pathway mutation associated changes across distinct genetic background. By overlapping mutation-driven genes between the two isogenic models, we identified 13 genes upregulated and 3 downregulated by *KRAS* mutation alteration, 16 upregulated and 53 downregulated by *BRAF* mutation alteration (Fig. 5A). Their expression changes across both cell lines are shown (Fig. 5B).

Given that *KRAS* and *BRAF* mutations are generally mutually exclusive [34], we next asked whether genes identified in cell models exhibited similar expression patterns in patient samples. To test this, we analyzed the U-CAN, TCGA COAD, and AC-ICAM cohorts and compared expression level between RAS pathway mutant and wild-type patients (Fig. 5C-D). Of all genes identified in the cell models, *ANO9*,* CHN2*,* GRM8*,* PPFIBP1*,* PPP2R3A*,* SEMA3A*,* SYBU*, and *ZNF488*, showed consistent direction of expression regulation across all three cohorts (Fig. 5E, Supplementary Fig. 7A). Since *BRAF* mutation is a well-established prognostic marker [6], we next examined whether any of these genes carried independent prognostic value. Stratification of patients by RAS pathway mutation status revealed that only in the U-CAN cohort did *BRAF* mutant patients show significantly worse overall survival compared to RAS wild-type patients (adjusted *p* = 0.0034) (Fig. 5F). By contrast, in the TCGA COAD and AC-ICAM cohorts, overall survival of *BRAF* mutant patients did not differ from either *KRAS* mutant or RAS wild-type patients (Supplementary Fig. 7B). We therefore focused subsequent survival analyses on U-CAN using the 13 genes that showed consistent expression pattern, among which *SLC2A1*,* SMAD3*,* PPFIBP1*, and *GRM8* showed prognostic significance (Supplementary Fig. 7C). As additional covariates such as age may also influence survival, we performed multivariate analysis, which revealed that only high *PPFIBP1* expression remained independently associated with poor overall survival (Fig. 5G).

### RUNX2 and TCF7L2 are associated with RAS pathway mutations in clinical cohorts

Since RUNX2 and TCF7L2 were identified in our cell models as TFs strongly linked to RAS pathway mutations, we next investigated whether they exhibited same role in clinical samples. We analyzed 81 publicly available ATAC-seq libraries derived from 41 CRC tissues. Among these, 6 samples were microsatellite instable (MSI; 2 *BRAF* mutant, 1 *KRAS* mutant, and 3 RAS wild-type), and 35 were microsatellite stable (MSS; 1 *BRAF* mutant, 11 *KRAS* mutant, 22 RAS wild-type, and 1 without reported mutation status). Due to the limited number of MSI tissues, we therefore pooled all samples and stratified them by RAS pathway mutation status for differential analysis.

**Fig. 6:**
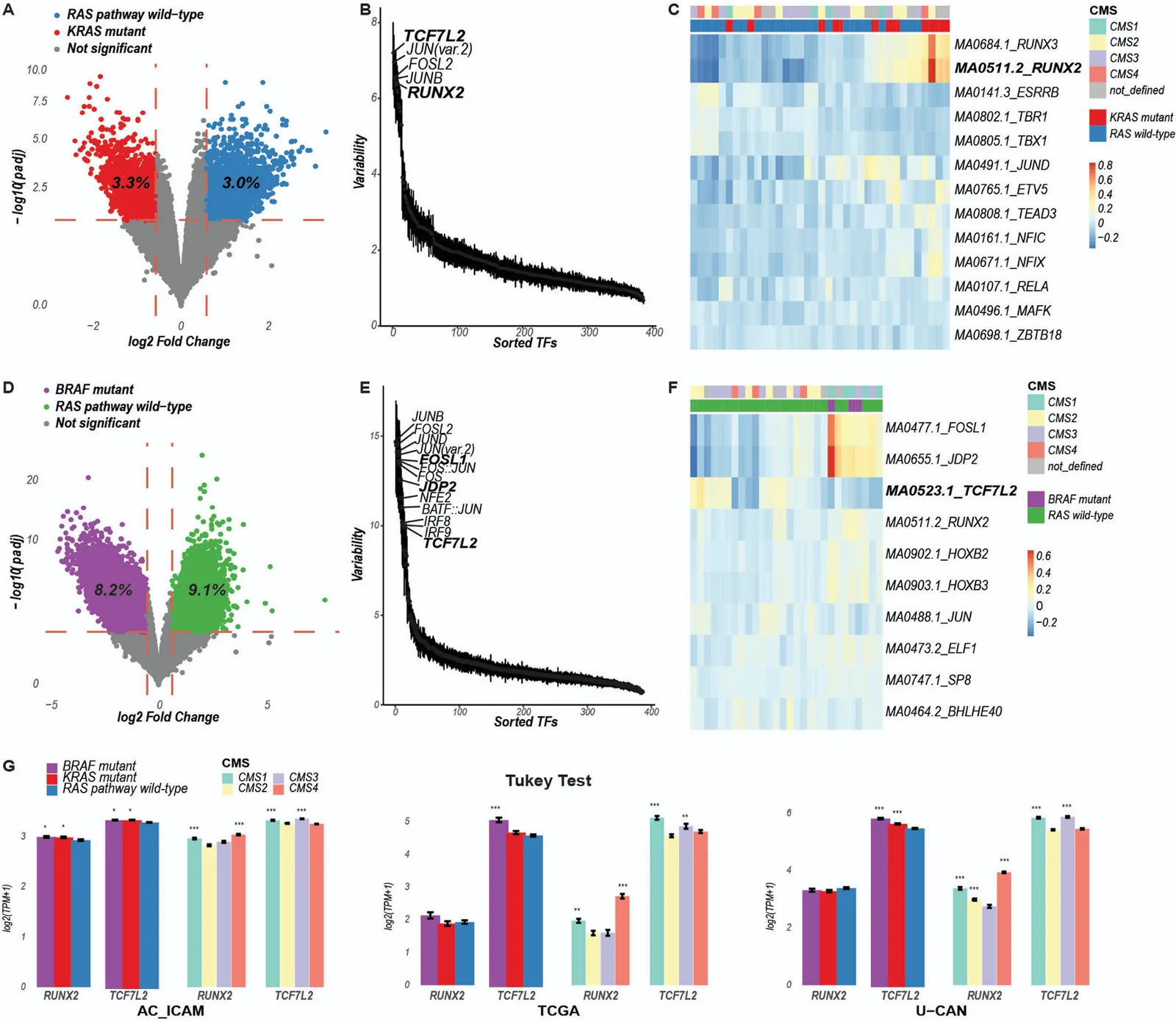
RUNX2 and TCF7L2 are TFs altered by RAS pathway mutations in clinical cohorts. **A** Volcano plot of regulatory regions associated with *KRAS* mutations in the TCGA COAD cohort (adjusted *p* < 0.05; |log2FC| > 0.58). **B** RUNX2 and TCF7L2 show variable binding activity when comparing *KRAS*-mutant to wild-type samples. **C** Heatmap of chromVAR deviation scores for TFs associated with *KRAS* mutation (*variability > 1*, adjusted *p* < 0.05), with patient mutation status and CMS indicated in the top annotation bars. **D** Volcano plot of regulatory regions associated with *BRAF* mutations in the TCGA COAD cohort (adjusted *p* < 0.05; |log2FC| > 0.58). **E** JDP2, FOSL1, and TCF7L2 display variable binding activity when comparing *BRAF*-mutant to wild-type samples. **F** Heatmap of chromVAR deviation scores for TFs associated with *BRAF* mutation (*variability > 1*, adjusted *p* < 0.05). **G** Bar plots of RUNX2 and TCF7L2 expression levels stratified by mutation status and CMS cluster across the AC-ICAM, TCGA, and U-CAN cohorts (*Tukey test*; significance indicated relative to the lowest group: p < 0.05*,p < 0.01**,p < 0.001***)

Among all regulatory regions identified from the TCGA COAD ATAC-seq dataset, 3.3% were associated with *KRAS*-mutant and 3.0% with wild-type samples (Fig. 6A). ChromVAR analysis identified TFs whose binding activity varied with mutation status, with TCF7L2 and RUNX2 ranking among the top 5 (Fig. 6B). T-tests further highlighted RUNX2 and RUNX3 most strongly associated with *KRAS* mutant CRCs (*p* < 0.05) (Fig. 6C). Applying the same approach to *BRAF* mutations, 8.2% of peaks were linked to *BRAF*-mutant and 9.1% to wild-type samples (Fig. 6D). The greatest variability was observed for AP1, IRF family members, and TCF7L2 (Fig. 6E). Deviation analysis additionally implicated FOSL1 and JDP2 in *BRAF*-mutant samples, while TCF7L2 showed preferential binding in wild-type samples (Fig. 6F). We then analyzed TF expression level with mutation from the AC-ICAM, TCGA COAD and U-CAN cohorts. ANOVA with Tukey’s post hoc test showed that *RUNX2* expression only slightly increases in mutants relative to wild type, while *TCF7L2* expression consistently increases in *BRAF* mutant patients. Stratification by consensus molecular subtype (CMS) revealed distinct expression patterns: *RUNX2* was strongly elevated in CMS4 and CMS1 relative to CMS3, while *TCF7L2* was highly expressed in CMS1 and CMS3 compared to CMS2 and CMS4 (Fig. 6G). Notably, when the CMS classification was applied to our clones, a significant shift in probability toward the CMS4 cluster was observed following *KRAS* mutation knockout in the HCT116 clones (Supplementary Fig. 8A).

At last, we assessed whether *RUNX2* and *TCF7L2* carried prognostic value. Log-rank analysis using the 75th percentile cutoffs demonstrated that high expression of both genes was associated with worse overall survival (Supplementary Fig. 8B-C). However, in multivariate model, prognostic significance was lost for *RUNX2* but retained for *TCF7L2* (Supplementary Fig. 8D-E).

## Discussion

Understanding how RAS pathway mutations drive CRC development remains a key challenge due to cancer heterogeneity. In this study, we used isogenic CRC models differing only in RAS pathway mutations and identified WNT signaling as a consistently regulated pathway at both epigenetic and transcriptional levels. Inferred TF results further highlighted RUNX2 and TCF7L2 as key regulators responsive to mutation alterations. Cross-comparison of associated changes across both cell models revealed 85 consistently altered genes, with TCF7L2 specifically linked to *BRAF* mutation. Notably, *SLC2A1* (GLUT1) emerged in *BRAF* mutant clones of both HCT116 and RKO, aligning with prior study validating it as a RAS mutation modulated gene [35], supporting its use as a positive marker in downstream analyses. To evaluate clinical relevance, we analyzed bulk ATAC-seq and RNA-seq data from multiple CRC cohorts, identified genes with concordant expression changes. RUNX2 and TCF7L2 were found associated with RAS pathway mutations in clinical cohorts. Although mutation-based stratification does not fully capture the regulatory changes observed in cell models, our results support the clinical relevance of these findings and underscore the value of isogenic systems for elucidating mutation-specific regulatory mechanisms in CRC.

WNT signaling appeared altered in response to RAS pathway mutations, its behavior differed between HCT116 and RKO cells. Based on the previously reported differences in β-catenin dependency [14], and the observation that WNT inhibition suppresses anchorage-independent growth in HCT116 but not in RKO [10, 11, 17], we hypothesized that these two cell lines differ in the interplay between RAS pathway mutations and WNT signaling. Specifically, HCT116 appears to be driven by both *KRAS* mutation and WNT signaling, while RKO exhibits WNT activation secondary to *BRAF* mutation. Recent single-cell studies suggest serrated polyps arise via mechanisms distinct from conventional adenomas that are driven by WNT-dependent stem cell expansion [36], potentially explaining the divergent mutation-driven WNT signaling seen in our cell models. Moreover, *BRAF* mutation-driven malignant transformation required months and was accompanied by CIMP acquisition, later shown to impact WNT signaling and differentiation genes [37]. Together, these findings indicate that WNT pathway activation is a consequence of *BRAF* mutation and age acquired methylation. Although *RNF43* mutations are common in MSI CRCs and often co-occur with *BRAF* mutations [6], previous study found WNT target genes suppression and loss of tumorigenic potential by CRISPR-correction of *BRAF* instead of *RNF43* mutation in CRC organoids [38], while *RNF43* mutations retain functionality and unlikely confer WNT-dependency onto CRCs [39].

Beyond WNT signaling, MYC is critical for maintaining cancer cell proliferation and stress adaptation [40, 41]. MYC is a well-established WNT/β-catenin target in *APC*-mutant CRC [42], this regulatory axis is unlikely to predominate in our models, as neither HCT116 nor RKO cells harbor *APC* mutations. Previous study reported that introduction of *KRAS* mutations in HCT116 upregulates MYC-target genes, while downregulated interferon-response genes [33, 43], consistent with our observations. Importantly, co-treatment with FDA-approved mTOR and proteasome inhibitors selectively eliminated *KRAS*-mutant CRC by disrupting MYC-driven stress adaptation [44], underscoring its *KRAS*-dependent role. Although the relationship between *BRAF* mutations and MYC in CRC is less studied, a recent CRISPR screen of epigenetic modifiers identified BRD2, an H3K27ac reader, as a vulnerability whose inhibition enhanced the therapeutic efficacy of combined BRAF and EGFR blockage. BET inhibition reduced H3K27ac levels at MYC-associated enhancers [45], including regions connecting MYC super-enhancers with FOSL1 binding sites [46]. Consistently, we observed increased FOSL1 binding patterns in *BRAF*-mutant (Supplementary Fig. 6F) CRCs. Moreover, MYC upregulation in RKO cells likely reflects a compensatory response to stress-induced MAPK signaling [47] and ETS-driven cellular plasticity [46] after *BRAF* mutation loss.

Given that mutation editing reshaped the epigenetic landscape in our models, we next sought to identify the upstream regulatory drivers. RUNX2 emerging as a key TF in HCT116 cells, consistent with previous reports [48]. In contrast, TCF7L2 was associated with *BRAF* mutations in both our isogenic models and patient cohorts. In our previous large CRC WGS and RNA-seq study, TCF7L2 was considered a tumor suppressor, with loss-of-function mutations frequently observed in non-hypermutated (MSS) CRC and no reported correlation with RAS pathway mutations [6]. In contrast, our current findings suggest that TCF7L2 expression changes primarily reflect WNT pathway activity and may support an oncogenic role. Notably, loss of TCF7L2 reduced proliferation in HCT116 and HT29 cells [10, 49], challenging its tumor suppressive function.

## Conclusions

Our findings show that although RAS pathway mutations induce robust and reproducible transcriptional and chromatin changes in isogenic models, these effects are often obscured in clinical tumors by additional sources of variability, including microsatellite instability, CpG island methylation, and co-occurring mutations. This underscores the value of isogenic systems for dissecting mutation-specific regulatory programs within a controlled genetic background. Insights from such models can guide the development of integrative frameworks that incorporate chromatin state and broader molecular context, ultimately enabling more precise patient stratification and therapeutic decision-making in CRC.

## Supplementary Information

Supplementary Material 1.

## Data Availability

ATAC-seq raw fastq data generated from this study has been submitted to European Genome Archieve (study ID: EGAS50000001757, dataset ID: EGAD50000002521).

## Abbreviations

CRC: Colorectal Cancer
TF: Transcription Factor
TSS: Transcription Start Site
CRE: Cis-Regulatory Element
PCA: Principal Component Analysis
TOBIAS: Transcription Factor Occupancy Prediction by ATAC-seq
chromVAR: Chromatin Variation Across Regions
U-CAN: Uppsala/Umeå Comprehensive Cancer Consortium
TCGA COAD: The Cancer Genome Atlas Colon Adenocarcinoma
AC-ICAM: Atlas and Compass of Immune-Cancer-Microbiome Interactions
CMS: Consensus Molecular Subtype
