# Supplementary material for: RAS pathway mutations orchestrate chromatin reconfiguration and transcriptional rewiring in colorectal cancer
Source: Cell Commun Signal. 2026 Oct 14;24:553. doi: 10.1186/s12964-026-03305-0 (PMC13663232; doi:10.1186/s12964-026-03305-0)
Supplement: Supplementary file 1 [file 12964_2026_3305_MOESM1_ESM.docx]

**Supplementary Material**

**Supplementary Figure 1: High quality and reproducible ATAC-seq data.** (A) Mutation status for *KRAS* and *BRAF* in isogenic models generated in previous ref^18^. (B) Total raw read counts for HCT116 harboring different RAS pathway mutations, with all samples passed 20 million reads (left). Fraction of reads in peaks (FRiP) for HCT116, with all samples passed 0.2 (right). (C) Total raw read counts for RKO harboring different RAS pathway mutations, with all samples passed 20 million reads (left). FRiP for RKO, with all samples passed 0.2. (D) Distribution of TSS enrichment scores for HCT116 and RKO clones. (E) Pairwise Pearson correlation heatmaps of ATAC-seq counts per million (CPM) for HCT116 clones (left) and RKO clones (right). (F) Pairwise Pearson correlation of heatmaps of RNA-seq transcripts per million (TPM) for HCT116 clones (left) and RKO clones (right). (G) Distribution of CRE features within ATAC-seq peak regions for HCT116 clones (top) and RKO clones (bottom).

**Supplementary Figure 2: Reintroduction of RAS pathway mutations in HCT116 clones modulates WNT/ β-catenin pathway, whereas RKO clones** **does not**. (A) *BRAF* mutation-associated genes and regulatory regions in HCT116, with enriched biological function. Volcano plot displays differentially expressed genes and regulatory regions (adjusted *p < 0.05*; *|log2FC| > 0.58*). Venn diagrams show intersecting genes between RNA-seq (top) and ATAC-seq (bottom). Heatmaps depict GO term clusters of intersecting genes (adjusted p < 0.05), with word clouds summarizing keywords for each cluster. (B) Bar plot showing the p-values of WNT related GO terms enriched in RAS pathway wild-type HCT116 clones (adjusted *p < 0.05*). (C) *KRAS* mutation-associated genes and chromatin regulatory regions in RKO clones, with enriched biological function. (D) Transcriptomic hallmark pathways regulated by *BRAF* mutation in HCT116 clones. Gene set enrichment analysis (GSEA) was performed using all expressed genes ranked by log2FC (adjusted *p < 0.05*). Pathways are shown as *BRAF* mutation upregulated (left) and downregulated (right). (E) Transcriptomic hallmark pathways regulated by *KRAS* mutation in RKO clones (adjusted *p < 0.05*), with upregulated (left) and downregulated (right) pathways.

**Supplementary Figure 3: TFs associated with RAS pathway mutations with RUNX2 and** **TCF7L2 consistently emerged as top TFs.**

(A) Heatmap showing significant TFs deviation associated with *KRAS* mutation in HCT116, identified by chromVAR (*variability > 1*; *t-test*, adjusted *p < 0.05*). (B) Heatmap showing significant TFs deviation associated with *BRAF* mutation in RKO (*variability > 1;* *t-test*, adjusted *p < 0.05*). (C) Volcano plot displaying significantly TFs footprint related to *KRAS* mutation in HCT116, identified by TOBIAS (adjusted *p < 0.05*). (D) Volcano plot displaying significantly TFs footprint related to *BRAF* mutation in RKO (adjusted *p < 0.05*).

**Supplementary Figure 4: Reintroduction of RAS pathway mutations into HCT116 RAS wild-type clones reveals emergence of TCF7L2 and** **RUNX2.** (A) *BRAF* mutation-associated CREs identified from differentially regulatory regions in HCT116. Top bar plot genomic distribution of TF-binding loci relative to TSS (-50 kb to +50 kb). Bottom bar plot CRE feature assignments of TF-binding loci, with promoters defined as 0-3 kb proximately to TSS. (B) *BRAF* mutation-associated TFs predicted by integrating binding loci accessibility and expression levels in HCT clones. Scatter plot (left) TF binding activity predicted using TOBIAS (adjusted *p < 0.05*) and chromVAR (*variability > 1, t-test* adjusted *p < 0.05*), with top 15 TFs highlighted. (C) *KRAS* mutation-associated CREs annotated in RKO. (D) *KRAS* mutation-associated TFs predicted by binding site accessibility (TOBIAS adjusted *p < 0.05*; chromVAR *variability > 1*, *t-test p < 0.05*) and expression integration (adjusted *p < 0.05*) in RKO.

**Supplementary Figure 5: TFs associated with RAS pathway with RUNX2 and TCF7L2** **emerged after *BRAF* mutation reintroduction in HCT116.**

(A) Heatmap showing significant TFs deviation associated with *BRAF* mutation in HCT116, identified by chromVAR (*variability > 1; t-test,* adjusted *p < 0.05*). (B) Heatmap showing significant TFs deviation associated with *KRAS* mutation in RKO (*variability > 1; t-test,* adjusted *p < 0.05*). (C) Volcano plot displaying significantly TFs footprint related to *BRAF* mutation in HCT116, identified by TOBIAS (adjusted *p < 0.05*). (D) Volcano plot displaying significantly TFs footprint related to *KRAS* mutation in RKO (adjusted *p < 0.05*).

**Supplementary Figure 6: TFs associated with RAS pathway mutations regulate downstream genes at both** **transcriptomic and epigenetic levels.** (A) Bar plots showing TFs that are upregulated (left) and downregulated (right) related to *KRAS* mutation in HCT116. TFs were identified using chromVAR and TOBIAS and mapped to their target genes using DoRothEA TF regulon collection. Only TF regulons with confidence levels A-D (supported by at least ChIP-seq evidence) were used. Intersecting target genes associated with RAS pathway mutations were used. (B) Bar plots of differentially regulated TFs related to *BRAF* mutation in HCT116. (C) Bar plots of differentially regulated TFs related to *BRAF* mutation in RKO. (D) Bar plots of differentially regulated TFs related to *KRAS* mutation in HCT116.

**Supplementary Figure 7: The expressions of *SMAD3*, *GRM8*, *SLC2A1*, and *PPFIBP1* are prognostic in U-CAN cohort.** (A) Expression of genes with consistent regulation by RAS pathway mutations in the AC-ICAM and TCGA COAD cohorts, with *SLC2A1* shown as a positive control. (B) KM overall survival plot stratified by RAS pathway mutation status in TCGA COAD (left) and AC-ICAM (right) cohorts. (C) KM overall survival plots of genes consistently regulated in both cell lines and clinical cohorts, with expression group defined by the 3rd quantile (75%).

**Supplementary Figure 8. Prognostic value of TCF7L2 in the U-CAN cohort independent of RAS pathway mutations and CMS subtypes.** (A) Prediction of CMS and Colorectal Prognostic Subtypes (CRPS) for HCT116 and RKO clones. Clones for which a subtype score exceeded 0.5 are marked with * to indicate their assigned subtype. (B) Kaplan–Meier (KM) overall survival plot stratified by *RUNX2* expression (3rd quartile, 75%) in the U-CAN cohort. (C) KM overall survival curve stratified by *TCF7L2* expression (3rd quartile, 75%). (D) Forest plot assessing *RUNX2* expression (3rd quartile, 75%) with adjustment for histology, pretreatment status, sex, tumor site, age group, mutation status, CMS subtype, tumor stage, and tumor grade. (E) Forest plot assessing *TCF7L2* expression (3rd quartile, 75%) with adjustment for histology, pretreatment status, sex, tumor site, age group, mutation status, CMS subtype, tumor stage, and tumor grade.

Supplementary Figure 9: *BRAF* V600E mutation acts as an independent driver of MAPK pathway activation. (A) Representative immunoblots showing levels of p-ERK1/2 and ERK1/2. β-actin was used as a loading control. (B) Quantification of band intensities or p-ERK 1/2 relative total ERK (n = 6) normalized to represented as mean +/- SD. Statistical analysis was performed by one-way ANOVA followed by Tukey’s multiple comparisons test (p < 0.05). (C) Expression levels of MAPK pathway-related genes (DUSP, MEK, and ERK) in HCT116 and RKO clones.
